# P17 A bend in the road: a patient with rheumatoid arthritis developing multicentric reticulohistiocytosis

**DOI:** 10.1093/rap/rkad070.038

**Published:** 2023-09-27

**Authors:** Rabia Firdous, Ganeshree Krishnan, Sara Sherif, Francesc Valenti, Chun Man Lee, Mark Lloyd

**Affiliations:** Frimley Park Hospital, Frimley, United Kingdom; Frimley Park Hospital, Frimley, United Kingdom; Frimley Park Hospital, Frimley, United Kingdom; Frimley Park Hospital, Frimley, United Kingdom; Frimley Park Hospital, Frimley, United Kingdom; Frimley Park Hospital, Frimley, United Kingdom

## Abstract

**Introduction:**

Multicentric reticulohistocytosis (MRH) is characterised by histiocyte and multicentric giant cell infiltration in the skin and joints, although any tissue can be involved. Classically, it presents with papulonodular skin eruptions and a rapidly erosive arthritis. Most cases are described in females in the fourth and fifth decades of life. The condition will often resolve over 10 years. Up to 25% of cases are associated with malignancy. Here we report a case of MRH presenting in a patient with RF, CCP positive rheumatoid arthritis (RA) diagnosed 10 years earlier. This is a rare disease combination and presents diagnostic and therapeutic challenges.

**Case description:**

A 56-year-old female presented in 2011 with a 2 year history of pain and swelling in the wrists, PIPs and ankle. Investigations: CRP 11, Hb 112, ferritin 10. TTG and endomysial antibodies positive. RF 125, CCP 33. X rays: radio-carpal joint space loss, right thumb IP joint erosion. Seropositive, erosive RA was diagnosed. She responded well to methotrexate (MTX) 20mg weekly. Patient declined endoscopy.

She was able to stop MTX in 2019 as symptoms had resolved. Hand X-rays showed no progression of erosions.

In December 2021, she presented with a month’s history of a rash on her hands with some associated right hand swelling and pain. The rash consisted of red macular areas on the dorsum of fingers and nodular elastic red confluent lumps especially on the dorsal distal phalanges and along the sides of the fingers. Several weeks later, an itchy rash appeared on the upper anterior trunk - macular with some areas of clustered discrete papular red lesions.

Epidermal biopsies showed reactive mild acanthosis and hyperkeratosis with sheets of histiocytes in the dermis and the presence of a Grenz zone. The histiocytes were negative for CD1a and S100 and positive for CD68. Findings were consistent with MRH.

CRP and CK were normal, Ro 52 positive, ANA and extended myositis panel negative. Hand X-rays showed no new erosions. CT chest, abdomen and pelvis and mammography showed no malignancy.

Prednisolone 30mg, MTX 20 mg weekly and hydroxychloroquine 200mg were started, with topical corticosteroids and alendronate 70mg weekly. The rash remained prominent, with wrist and MCP swelling. Adalimumab was started in July 2022. Prednisolone has been weaned to 5mg alternate days but she has required several courses of higher doses. X-rays in June 2023 show probable bilateral index finger DIP erosions but joint and skin symptoms have largely resolved.

**Discussion:**

The long period between initial presentation and subsequent development of MRH in our patient suggests she has two separate conditions. However, it is possible that previous treatment had kept the MRH at bay. The presence of DIP erosions suggests that MRH may now be the predominant arthropathy. In MRH joint symptoms precede cutaneous disease in up to 60% of cases, as a symmetric polyarthritis affecting hands (PIPs particulalry, DIPs, MCP), shoulders, knees, wrists, hips, elbows, ankles and feet. Erosions can occur rapidly. Inflammatory markers tend to be normal.

Skin lesions consist of pink or reddish brown papulo-nodules, up to 2cm diameter, involving the upper body, acral and extensor surfaces, dorsum of the fingers, and nail folds. A “coral bead” appearance is considered pathognomonic, though present in < 50% cases. Skin lesions occasionally join to form large plaques. They rarely ulcerate or leave scars. Mucosal lesions have also been described in some cases. Biopsy is diagnostic.

MRH is rare. The largest recent single case series is from the Mayo Clinic in 2020 and includes 24 patients. Interestingly, 25% of these patients had auto-immune conditions, including 3 with RA. 5 had positive RF, 8 had positive CCP. The most common associated malignancy was melanoma.

We have found reports of 7 cases of concomitant RA and MRH; generally RA appears to occur before the MRH. In 3 of these cases, symptoms resolved with combinations of MTX, corticosteroids and bisphosphonates. Otherwise, treatment of MRH is essentially empirical. MTX, corticosteroids and bisphosphonates are commonly used first line, with a range of anti-rheumatic monoclonal agents also being reported in the literature. More recently there have been reports of successful treatment with jakinibs. However, the rapid progression of erosive arthritis in some cases means that patients can be left with damaging arthritis.

**Key learning points:**

Our case highlights the importance of diagnosing MRH, a rare but potentially destructive cause of arthritis. The clinical suspicion of MRH should be raised in patients with characteristic skin and joint involvement as MRH can easily be misdiagnosed as other systemic rheumatological conditions. In particular, RA presents in the same age and sex group, and positive rheumatoid serology appears to be relatively common in MRH. Psoriatic arthropathy can also be a challenging differential diagnosis, with skin lesions and erosive DIP change. As MRH can present with joint symptoms alone, a low threshold of diagnostic suspicion is needed.

Once diagnosed, patients should be screened for malignancy. Patients need to be referred urgently to secondary care so that early treatment can be started to try to prevent joint damage. Appropriate sensitive patient counselling and education is necessary, given the variable responses to treatment and variable course. Close collaboration between dermatology and rheumatology is important.

The coexistence of RA and MRH can make assessment of joint inflammation and treatment choice difficult. However, it also opens up potential dual disease treatment pathways. We suggest the rarity and severity of MRH make it a suitable candidate for a national registry, jointly managed between rheumatology and dermatology. This would increase awareness of the condition and enable better informed decisions regarding treatment.

